# Colon capsule endoscopy versus CT colonography in FIT-positive colorectal cancer screening subjects: a prospective randomised trial—the VICOCA study

**DOI:** 10.1186/s12916-020-01717-4

**Published:** 2020-09-18

**Authors:** Begoña González-Suárez, Mario Pagés, Isis Karina Araujo, Cristina Romero, Cristina Rodríguez de Miguel, Juan Ramón Ayuso, Àngels Pozo, Maria Vila-Casadesús, Anna Serradesanferm, Àngels Ginès, Glòria Fernández-Esparrach, Maria Pellisé, María López-Cerón, David Flores, Henry Córdova, Oriol Sendino, Jaume Grau, Josep Llach, Miquel Serra-Burriel, Andrés Cárdenas, Francesc Balaguer, Antoni Castells

**Affiliations:** 1 Gastroenterology Department, Hospital Clinic of Barcelona, Barcelona, Spain; 2 Centro de Investigación Biomédica en Red de Enfermedades Hepáticas y Digestivas (CIBEREHD), Madrid, Spain; 3 Radiology Department, Hospital Clinic of Barcelona, Barcelona, Spain; 4 Institut d’Investigacions Biomèdiques August Pi i Sunyer (IDIBAPS), Universitat de Barcelona, Barcelona, Catalonia Spain; 5 Department of Preventive Medicine and Epidemiology, Hospital Clinic of Barcelona, Barcelona, Spain; 6 Center for Research in Health and Economic, Pompeu Fabra University, Barcelona, Spain

**Keywords:** Colon capsule endoscopy, CT colonography, Colorectal cancer screening

## Abstract

**Background:**

Colon capsule endoscopy (CCE) and CT colonography (CTC) are minimally invasive techniques for colorectal cancer (CRC) screening. Our objective is to compare CCE and CTC for the identification of patients with colorectal neoplasia among participants in a CRC screening programme with positive faecal immunochemical test (FIT). Primary outcome was to compare the performance of CCE and CTC in detecting patients with neoplastic lesions.

**Methods:**

The VICOCA study is a prospective, single-centre, randomised trial conducted from March 2014 to May 2016; 662 individuals were invited and 349 were randomised to CCE or CTC before colonoscopy. Endoscopists were blinded to the results of CCE and CTC.

**Results:**

Three hundred forty-nine individuals were included: 173 in the CCE group and 176 in the CTC group. Two hundred ninety individuals agreed to participate: 147 in the CCE group and 143 in the CTC group. In the intention-to-screen analysis, sensitivity, specificity and positive and negative predictive values for the identification of individuals with colorectal neoplasia were 98.1%, 76.6%, 93.7% and 92.0% in the CCE group and 64.9%, 95.7%, 96.8% and 57.7% in the CTC group. In terms of detecting significant neoplastic lesions, the sensitivity of CCE and CTC was 96.1% and 79.3%, respectively. Detection rate for advanced colorectal neoplasm was higher in the CCE group than in the CTC group (100% and 93.1%, respectively; RR = 1.07; *p* = 0.08). Both CCE and CTC identified all patients with cancer. CCE detected more patients with any lesion than CTC (98.6% and 81.0%, respectively; RR = 1.22; *p* = 0.002).

**Conclusion:**

Although both techniques seem to be similar in detecting patients with advanced colorectal neoplasms, *CCE* is *more sensitive* for the *detection* of any neoplastic lesion.

**Trial registration:**

ClinicalTrials.gov Identifier: NCT02081742. Registered: September 16, 2013.

## Background

**What is known?**

- Colorectal cancer (CRC) is the fourth leading cause of cancer death worldwide and the second in Europe. The introduction of minimally invasive methods for colorectal cancer diagnosis is recommended in order to increase patient’s adherence.
- Colon capsule endoscopy and CT colonography are two minimally invasive techniques with high sensitivity and specificity in detecting colorectal cancer. No comparative and prospective studies have been done between both tests so far.

**What is new here?**

- Colon capsule endoscopy detected more patients with any neoplastic lesion (regardless of size).
- Colon capsule endoscopy is superior to CT colonography for detecting patients with significant lesions (i.e. ≥ 6 mm in size at colonoscopy), with a slightly lower specificity.
- Colon capsule endoscopy and CTC are well-accepted, useful and safe strategies, with similar performance in terms of advanced neoplasm detection rate. However, CCE may benefit from a higher sensitivity for detecting small, flat, sessile and serrated lesions. The impact of these results in terms of overall effectiveness deserves further investigation.

Colorectal cancer (CRC) is the fourth leading cause of cancer death worldwide and the second in Europe. Over the last decade, mortality has decreased due to the introduction of new treatments and screening programmes [1, 2]. Indeed, clinical guidelines recommend screening in average-risk (individuals over the age of 50 who have no additional risk factors) and high-risk (i.e. hereditary CRC syndromes, individuals with first-degree relatives with CRC, or patients with inflammatory bowel disease) populations.

There are different strategies for CRC screening, but the most extensively accepted are colonoscopy, flexible sigmoidoscopy and faecal occult blood testing (i.e. faecal immunochemical tests [FIT]). Other screening methods include CT colonography (CTC), colon capsule endoscopy (CCE) and DNA tests [3]. In a randomised, controlled trial conducted by our group, subjects randomised to FIT were more likely to participate in screening than those randomised to colonoscopy. Moreover, on the baseline screening exam, a similar number of patients with CRC were detected in each study arm, but more adenomas were identified in the colonoscopy group [4].

In FIT-positive subjects, colonoscopy is the second step to confirm colorectal neoplastic lesions [5]. However, colonoscopy shows a variable risk of complications (i.e. bleeding or perforation), leading to a low participation rate. Moreover, in up to 10% of individuals, caecal intubation is not achieved, and an alternative imaging technique is needed [6]. In this context, CTC and CCE have been shown to be valid procedures for such a purpose [7–9]. Indeed, CTC is a radiological technique widely accepted by patients, with a high sensitivity and specificity in detecting significant colorectal lesions [10]. Major limitations are the radiation amount which the individual is exposed to and its ability to detect small and flat lesions [10]. On the other hand, CCE is a minimally invasive and safe method of visualising the entire colon and represents an alternative for CRC screening and diagnosis [11, 12]. The second-generation capsule has demonstrated high sensitivity and specificity in the detection of patients with polyps [5, 11, 13, 14]. CCE limitations include the intensive laxative preparation needed and time required to read every study (40–50 min, approximately). Importantly, both strategies have never been compared in a parallel manner in order to determine their specific role in the CRC diagnostic algorithm.

The aim of the present study was to compare CCE and CTC for the identification of patients with colorectal neoplasia among participants in a population-based, organised CRC screening programme who had a FIT-positive result. This study design allows us to evaluate an enriched population with high prevalence of colorectal neoplasms [5].

## Methods

### Study design and population

The VICOCA study is a prospective, single-centre, randomised trial (NCT02081742) conducted in a tertiary referral hospital in Barcelona. Enrolment began in March 2014 and ended in May 2016. The study was approved by the institutional ethics committee. All authors had access to the study data, reviewed and approved the final manuscript.

The primary outcome was to compare performance characteristics (i.e. sensitivity, specificity, positive and negative predictive values and overall accuracy) of CCE and CTC in detecting patients with colorectal neoplastic lesions, using colonoscopy as an enhanced gold standard (segmental unblinding has been used to increase the accuracy of our evaluation [15]) in a FIT-positive screening population.

The secondary outcomes were (1) to compare advanced colorectal neoplasm detection rate of CCE and CTC if a threshold of ≥ 6 mm in size was used to indicate the work-up colonoscopy; (2) to determine the false-positive and false-negative rates of CCE and CTC in detecting significant lesions (i.e. ≥ 6 mm in size at colonoscopy), and to identify predictive factors for false-negative results of either CCE or CTC; and (3) to determine the incidence of adverse events associated with each screening strategy.

Individuals with a positive FIT result (≥ 20 μg of haemoglobin/g of faeces) from the population-based, organised CRC screening programme of Barcelona, which targets men and women aged 50 to 69, were eligible for the study. Subjects with symptoms, personal history of inflammatory bowel disease, colorectal adenomas, CRC, or total/partial colectomy, or family history of colorectal polyposis or other inherited disorders, were excluded from the screening programme, and therefore, they were not eligible for the study. Individuals with any contraindication for CCE and CTC, such as suspected intestinal stricture or allergy to study drugs, were excluded from the study.

All eligible individuals without exclusion criteria signed an informed consent form at the local screening office and were randomly allocated to CCE or CTC through a computer-generated list. Sealed opaque envelopes were created by an external nurse not participating in the study. Colonoscopy was performed 1–2 weeks after these procedures and used as the gold standard in both groups. For correct anatomical correlation of findings of each examination, the colon was divided into six segments: caecum, ascending colon, transverse colon, descending colon, sigmoid and rectum.

After colonoscopy, any relevant therapeutic procedures were undertaken if necessary. Subsequently, patients were followed at an outpatient clinic to detect any potential adverse event appearing within 30 days of colonoscopy.

### Colon capsule endoscopy

Colon capsule retrieves images of the entire colon in a minimally invasive manner without the need for insufflation. It is a 11 × 33 mm long device swallowed by the patient that traverses the entire digestive tube with the help of normal peristaltic movements [16].

The second-generation CCE used in this study (PillCam® COLON2 Capsule Endoscopy; Medtronic, Minneapolis, MN) has two cameras, one at each end, with a 172-degree angle in each camera. The Rapid software, versions 7 and 8, includes a ‘polyp size estimation software’ that calculates the polyp length by moving the cursor from one end of the polyp to the other [11, 12].

The CCE preparation included a low-volume laxative solution (Moviprep®: PEG-3350, sodium sulphate, sodium chloride, potassium chloride, sodium ascorbate and ascorbic acid for oral solution; Norgine B. V, UK) combined with Gastrografin® (diatrizoate meglumine and diatrizoate sodium solution USP; Berlimed SA, Madrid, Spain) [15] (Table S1). The cleansing level was evaluated based on a previously validated scale [17, 18] and classified as poor (large amount of faecal residue), fair (enough residue to preclude a completely reliable examination), good (small amount of residue, not enough to interfere with examination) and excellent (no more than small bits of adherent faeces) for each colonic segment. Examinations scored as ‘poor’ or ‘fair’ in any segment were considered ‘inadequate’, whereas those scored as ‘good’ or ‘excellent’ in all segments were considered ‘adequate’.

Lesions identified by CCE were classified according to their morphology as sessile, pedunculated or flat. All videos and images were reviewed by two expert CCE readers (BGS and IKA) before colonoscopy.

### CT colonography

CT colonography obtains 2D and 3D images of the colon through CT. It works by insufflating air or CO_2_ into the colon through a flexible rectal cannula to achieve appropriate distension. In this study, a 64-Chanel CT scanner (Sensation; Siemens, Germany) was used. No intravenous contrast agent was administered during the procedure to any patient, except to perform the staging when a CRC was detected.

In preparation for the CTC, no laxative was used [19–21]. Individuals were required to consume a non-fibre diet and 7.5 ml of Gastrografin® diluted in water 2 days before the CTC (five doses per day at breakfast, mid-morning, lunch, afternoon and dinner). Taking 200 ml of Nutrison standard® (Nutricia, Ireland) as a diet supplement was deemed optional. Drinking a large quantity of water was also recommended during the preparation.

Colonic distension was carried out using CO_2_ from an automated insufflator (PROTOCO_2_L**®** colon insufflator, E-Z-EM, Monroe Township, NJ). A scout view of the abdomen was obtained once signs of a distended colon were evident, and CT was then performed in prone position. This procedure was repeated in supine position. The analysis of images was achieved by 2D primary reading, using 3D as problem solving. If lesions suggesting a malignant nature were detected in the first acquisition (prone position), intravenous contrast was administered for the second one (supine) and an appropriate extension study was carried out. The maximum duration of the procedure was 20 min and was done with low radiation dose and non-cathartic preparation.

The quality of examination was classified as ‘adequate’, partial or inadequate based on three parameters: amount of residue in the colon lumen, distension of the colon and faecal tagging.

According to their morphology, lesions were classified as sessile, pedunculated and flat. They were measured in the cross section where the largest diameter was found. An expert radiologist (MP), with over 10 years of experience in this technique, examined all CTC readings before colonoscopy.

### Colonoscopy

All colonoscopies were performed under deep sedation overseen by an anaesthesiologist and after proper bowel preparation. Colonoscopy was carried out by experienced endoscopists with a global adenoma detection rate of 54.9% in FIT-based screening [4], who were blinded to the results of prior examinations (i.e. CCE and CTC). The cleansing level was evaluated according to the Boston Bowel Preparation Scale [17]. Examinations with a total score < 6 points, or < 2 points in any segment, were considered inadequate, and therefore, they were repeated. The Paris classification [18, 22] was used to describe the identified lesions.

All lesions detected during colonoscopy were removed and/or biopsied following the usual protocol to obtain a histological description of each lesion. Advanced colorectal neoplasm was defined as invasive cancer, advanced adenoma or advanced serrated lesions. Adenomas ≥ 10 mm in size, with villous architecture, high-grade dysplasia or intramucosal carcinoma were classified as advanced adenomas. Serrated lesions ≥ 10 mm in size or with dysplasia were considered advanced serrated lesions. Invasive cancer was considered when malignant cells were observed beyond the muscularis mucosa. Tumour staging was performed according to the AJCC classification. Patients were classified according to the most advanced lesion [4].

### Lesion matching

During colonoscopy, the endoscopist who read the CCE or the radiologist who carried out the CTC remained at the examination room for a segmental unblinded revision. Indeed, if a lesion measuring ≥ 6 mm in diameter was seen on CCE or CTC but not in the initial colonoscopy, the endoscopist re-examined the corresponding colonic segment.

A lesion-matching algorithm was used to address inherent uncertainties in the comparison of localisations and sizes. For a given lesion to be considered a true positive match between CCE or CTC and colonoscopy, it has to be assessed as appearing within the same colonic segment or in adjacent segments, and the two recorded diameters had to be the same, with a 50% margin of error [23]. In case of discrepancies, an independent panel of experts (constituted by one endoscopist, one radiologist and one capsule reader) made the final decision based on the photographs and CT images of the identified lesions. Lesions observed by CCE or CTC, and not identified later by colonoscopy, were considered as a false-positive result of the first examination. When this circumstance occurred, colonoscopy was repeated only if there was a high degree of suspicion of a missing lesion.

### Sample size and power calculation

As mentioned before, this study was aimed at comparing the performance of CCE and CTC for the identification of patients with colorectal neoplasia among FIT-positive participants in a population-based, organised CRC screening programme, which constitute an enriched population for colorectal neoplasms (i.e. prevalence of neoplastic lesions over 60% [4, 24]). In such a context, it was assumed that CCE would detect a similar number of lesions as the gold standard, whereas a difference ≥ 15% between CCE and CTC would be considered clinically significant. Accordingly, sample size was estimated in 173 individuals per group, with a 5% level of significance and a statistical power of 80%.

### Statistical analysis

Study outcomes were assessed by intention-to-screen (main analysis) and per-protocol (secondary analysis). While the former analysis included all individuals who complete the study (i.e. colonoscopy), the latter was limited to those completing the study in whom CCE and CTC was fully evaluable.

Performance characteristics of CCE and CTC in detecting patients with colorectal neoplastic lesions, using colonoscopy as the gold standard, include sensitivity, specificity, positive and negative predictive values and overall accuracy. These figures were calculated considering both patients with any neoplastic lesion detected at colonoscopy and only those in whom neoplastic lesions ≥ 6 mm or ≥ 10 mm in size measured at colonoscopy were found, with a 95% confidence interval.

On the other hand, the detection rate was calculated as the number of individuals in whom colorectal lesions (i.e. cancer, advanced neoplasm or any neoplastic lesion) were detected in each study arm with respect to the number of patients in whom the corresponding lesions were identified at colonoscopy, if a threshold of ≥ 6 mm in size of lesions detected by either CTC or CCE was used to indicate the work-up colonoscopy.

The chi-squared test was used to compare categorical variables and Student’s *t* test for continuous variables. Independent predictors for false-negative results of either CCE or CTC were ascertained in a multivariable logistic regression analysis including individual’s demographics (gender and age), polyp characteristics (size, location and histology) and imaging quality parameters of both techniques.

A *p* value < 0.05 was considered statistically significant in all analyses. The IBM SPSS package (version 21.0) was used for the statistical analysis.

## Results

**Fig. 1:**
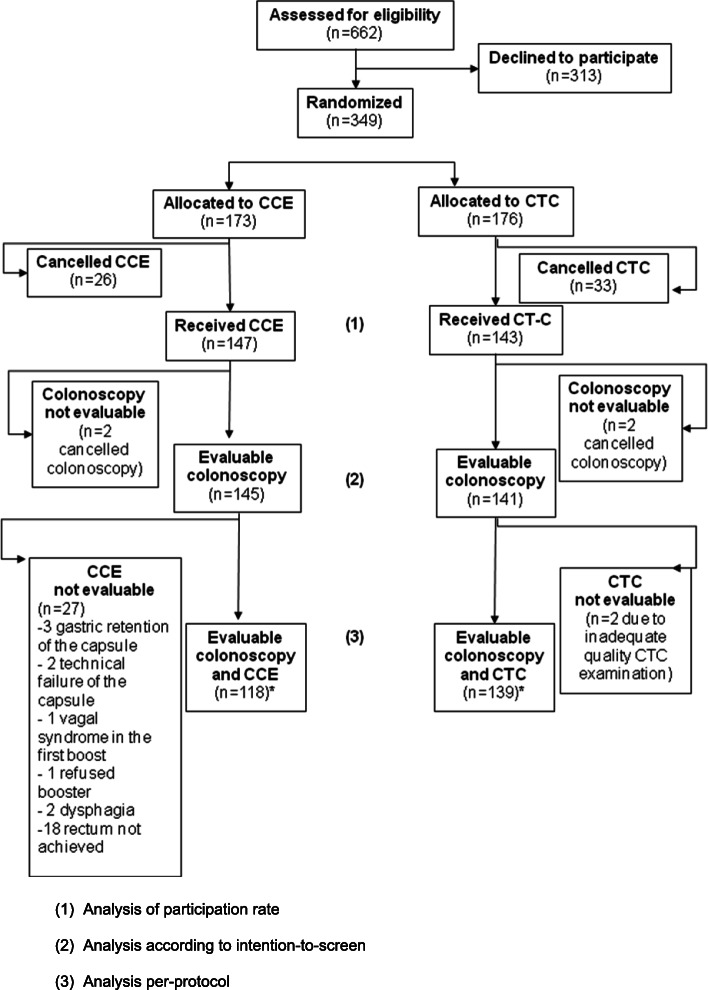
Flow chart of the study

Six-hundred and sixty-two FIT-positive participants in the CRC Screening Programme of Barcelona were eligible for this study. Three-hundred and forty-nine individuals agreed to participate, signed the informed consent form and were included in the study. The flow chart of the study is depicted in Fig. 1. As it is shown, 313 out of 662 subjects (47.2%) declined to participate in the study, there being no differences with those who actually did in terms of demographic characteristics and detected lesions (Table S2). The main reason for non-acceptance to participate was the lack of time to perform the study tests.

Twenty-six individuals of the CCE group and 33 individuals of the CTC group cancelled the examination, there being no difference in acceptance between both groups [CCE, 147 (84.9%) individuals; CTC, 143 (81.3%) individuals; *p* = 0.35]. In addition, two individuals in each group did not complete colonoscopy, and therefore, they were not considered in all remaining analyses. Therefore, 286 individuals were included in the intention-to-screen analysis (50% female; mean age, 60.1 ± 5.7 years old): 145 in the CCE group and 141 in the CTC group. Finally, 27 individuals in whom CCE could not be adequately evaluated and 2 subjects with inadequate quality CTC examination were not included in the per-protocol analysis (Fig. 1).

Table 1 shows the demographic characteristics and colonoscopy findings of individuals included in the intention-to-screen analysis. All colonoscopies were complete (i.e. examination reached the caecum). Sixteen (5.6%) colonoscopies were repeated due to inadequate preparation and the combination of both examinations was used as the gold standard for the study. Overall, 98.9% of colonoscopies had an acceptable bowel preparation. The ascending colon cleansing level was adequate in 99.6% of patients, transverse colon in 99.6% and descending-sigmoid and rectum in 99.3% of patients. Lesions were detected in 206 out of 286 (72.0%) individuals: 135 (47.2%) had lesions ≥ 6 mm, 77 (26.9%) had lesions ≥ 10 mm and 12 (4.1%) had CRC. As it is shown in Table 1, there were no significant differences between study groups regarding lesions detected at colonoscopy.

**Table 1:** Demographic characteristics and colonoscopy findings of individuals included in the intention-to-screen analysis

	CCE group (*n* = 145)	CTC group (*n* = 141)	*p* value
Demographics			
Age (years old)^1^	60.1 (5.8)	60.0 (5.9)	0.79
Gender			0.05
Male	83 (57.2%)	64 (45.4%)	
Female	62 (42.8%)	77 (54.6%)	
Findings at colonoscopy			
Repeat colonoscopy			0.74
No	138 (95.2%)	131 (93.6%)	
Yes	7 (4.8%)	9 (6.4%)	
Number of lesions^1^	2.7 (3.7)	2.1 (3.4)	0.15
Any lesion^2^ (regardless of size)			0.06
No	33 (22.8%)	47 (33.3%)	
Yes	112 (77.2%)	94 (66.7%)	
Any lesion ≥ 6 mm			0.06
No	68 (46.9%)	83 (58.9%)	
Yes	77 (53.1%)	58 (41.1%)	
Any lesion ≥ 10 mm			0.35
No	108 (74.5%)	101 (71.6%)	
Yes	37 (25.5%)	40 (28.4%)	
Invasive cancer			0.35
No	141 (97.2%)	133 (94.3%)	
Yes	4 (2.8%)	8 (5.7%)	
Advanced neoplasm^3^			0.44
No	102 (70.3%)	97 (68.7%)	
Yes	43 (29.7%)	44 (31.2%)	
Serrated lesions			0.68
No	108 (74.5%)	109 (77.3%)	
Yes	37 (25.5%)	32 (22.7)	

*CCE* colon capsule endoscopy, *CTC* CT colonography

^1^Continuous variables are expressed as mean (standard deviation)

^2^Any neoplastic lesion includes cancer, advanced and non-advanced adenomas, and advanced and non-advanced serrated lesions

^3^Advanced neoplasm includes invasive cancer, advanced adenomas and advanced serrated lesions

### Diagnostic performance

In the CCE group, adequate preparation (excellent or good prep) was achieved in 118 out of 145 individuals (81.7%). In the intention-to-screen analysis, sensitivity, specificity and positive and negative predictive values of CCE examination for the detection of patients with any neoplastic lesion were 98.1%, 76.6%, 93.7% and 92.0%, respectively (Table 2). Two patients (1.4%) presented lesions at colonoscopy not detected by CCE (false-negative results): one patient had two 4-mm sessile polyps in the sigmoid and one 8-mm pedunculated polyp in the rectum, whereas the second patient had two 2-mm and 3-mm sessile polyps close to the appendicular orifice. In the first patient, CCE examination was incomplete due to capsule deactivated at the transverse colon because the battery ran out. On the other hand, colonoscopy did not identify any lesion in 7 patients in whom CCE reported 8 lesions, 6 of them ≥ 6 mm in size (false-positive results).

**Table 2:** Diagnostic performance (all figures are expressed as percentages) of colon capsule endoscopy and CT colonography, according to the intention-to-screen analysis

	CCE	95% CI	CTC	95% CI	Diff.	95% CI
Any neoplastic lesion^1^ (regardless of size), no. (%)	112 (77.2)^2^		94 (66.7)^2^			
Sensitivity	98.1	[94.1; 99.4]	64.8	[56.7; 72.2]	33.2	[22.8; 43.0]
Specificity	76.6	[68.8; 82.9]	95.7	[91.0; 98.0]	− 19.1	[− 35.6; − 2.4]
PPV	93.6	[88.2; 96.7]	96.8	[92.4; 98.7]	− 3.1	[− 9.5; 3.9]
NPV	92.0	[86.2; 95.4]	57.6	[49.4; 65.5]	34.3	[16.9; 48.7]
Accuracy	93.3	[87.9; 96.4]	75.1	[67.4; 81.5]	18.2	[9.7; 26.4]
Any neoplastic lesion^1^ ≥ 6 mm, no. (%)^3^	77 (53.1)^2^		58 (41.1)^2^			
Sensitivity	96.1	[91.1; 100]	79.3	[68.6; 88.8]	16.7	[5.2; 28.2]
Specificity	88.2	[79.6; 95.3]	96.3	[91.1; 100]	− 8.1	[− 17.1; 0.1]
PPV	90.2	[83.5; 96.1]	93.8	[85.7; 100]	− 3.6	[− 12.9; 6.5]
NPV	95.2	[89.2; 100]	86.9	[80.0; 93.2]	8.2	[0.9; 16.9]
Accuracy	92.4	[87.5; 96.5]	89.3	[83.6; 93.6]	3	[− 3.7; 9.8]
Any neoplastic lesion^1^ ≥ 10 mm, no. (%)^3^	37 (25.5)^2^		40 (28.4)^2^			
Sensitivity	97.3	[91.1; 100]	90.0	[83.9; 93.9]	7.3	[− 4.5; 18.6]
Specificity	95.3	[90.7; 99.0]	99.0	[95.6; 100]	− 3.6	[− 8.3; 1.2]
PPV	87.8	[76.7; 97.3]	97.3	[93.1; 100]	− 9.5	[− 21.2; 2.9]
NPV	99.0	[96.8; 100]	96.1	[91.5; 98.2]	2.8	[− 1.6; 7.4]
Accuracy	95.5	[92.4; 99.3]	96.4	[91.9; 98.4]	− 0.8	[− 5.2; 4.0]

*CCE* colon capsule endoscopy, *CTC* CT colonography, *95% CI* 95% confidence interval, *PPV* positive predictive value, *NPV* negative predictive value, *Diff.* difference

^1^Any neoplastic lesion includes cancer, advanced and non-advanced adenomas, and advanced and non-advanced serrated lesions

^2^Prevalence of patients with such lesions at colonoscopy

^3^Lesion size was estimated at colonoscopy

The quality of CTC was considered adequate in 122 out of 141 individuals (86.6%) based on colon distension, faecal labelling and absence of faecal residues. In the intention-to-screen analysis, sensitivity of CTC examination for the detection of patients with any neoplastic lesion was 64.9%, with specificity and positive and negative predictive values of 95.7%, 96.8% and 57.7%, respectively (Table 2). Thirty-three patients (23.4%) presented lesions at colonoscopy not detected by CTC (false-negative results), 11 out of them (7.8%) with lesions ≥ 6 mm (three of them larger than 10 mm, 2 sessile polyps and 1 flat polyp). On the other hand, 2 patients undergoing CTC had one polyp (5-mm sessile polyp in descending colon and 8-mm sessile polyp in sigmoid, respectively) that was overlooked at colonoscopy (false-positive results).

In terms of detecting patients with significant lesions (i.e. ≥ 6 mm in size at colonoscopy), the sensitivity of CCE and CTC was 96.1% and 79.3%, respectively (*p* = 0.0003), whereas the corresponding figures for specificity were 88.2% and 96.3% (*p* = 0.03), respectively (Table 2).

The results of the per-protocol analysis are shown in Table S3. Indeed, after excluding those individuals in whom either CCE (27 subjects) or CTC (2 subjects) was not fully evaluable (Fig. 1), values of 100% and 80% for sensitivity of CCE and CTC, respectively, were observed in detecting patients with significant lesions (*p* < 0.00001).

### Neoplasm detection rate

Using a threshold of ≥ 6 mm in size of lesions detected by either CCE or CTC, we observed that the detection rate for advanced colorectal neoplasm in the CCE group was higher than that in the CTC group, but this difference did not achieve statistical significance (100% (43 out of 43 patients) and 93.1% (41 out of 44 patients), respectively; RR = 1.07; *p* = 0.08) (Table 3). Both CCE and CTC identified all patients with cancer, while CCE detected more patients with any neoplastic lesion than CTC ((74 out of 75 patients) 98.6% and (47 out of 58 patients) 81.0%, respectively; RR = 1.22; *p* = 0.002) (Table 3).

**Table 3:** Detection rate (the detection rate was calculated as the number of individuals in whom colorectal lesions (i.e. cancer, advanced neoplasm or any neoplastic lesion) were detected in each study arm with respect to the number of patients in whom the corresponding lesions were identified at colonoscopy, if a threshold of ≥ 6 mm in size of lesions detected by either CTC or CCE was used to indicate the work-up colonoscopy) of colon capsule endoscopy and CT colonography, according to the intention-to-screen analysis

Colorectal lesion^**1**^	CCE	CTC	RR	95% CI	***p*** value
Cancer	4 (100%)	8 (100%)	1	1.00–1.00	–
Advanced neoplasm^2^	43 (100%)	41 (93.1%)	1.07	0.99–1.16	0.08
Any neoplastic lesion^3^	74 (98.6%)	47 (81.0%)	1.22	1.07–1.38	0.002

*CCE* colon capsule endoscopy, *CTC* CT colonography, *RR* relative risk, *95% CI* 95% confidence interval

^1^Patients were classified according to the most advanced lesion

^2^Advanced neoplasm includes invasive cancer, advanced adenomas and advanced serrated lesions

^3^Any neoplastic lesion includes cancer, advanced and non-advanced adenomas, and advanced and non-advanced serrated lesions

Upon excluding individuals in whom either CCE or CTC was not fully evaluable (per-protocol analysis), results did not vary meaningfully (Table S4).

### Analysis at lesion level

For the analysis at lesion level, three patients were not evaluable due to the large number of lesions found at colonoscopy (Fig. 1), which made it difficult to determine a precise correlation with CCE and CTC results.

As it is shown in Table 4, a higher number of lesions were detected by CCE compared to CTC 83.2% (298 out of 358 lesions) and 42.8% (119 out of 278 lesions), respectively (*p* < 0.001). This difference is mainly due to a higher capability of CCE for detecting small lesions and lesions with sessile or flat morphology, in comparison with CTC (Table 4).

**Table 4:** Sensitivity (all figures are expressed as percentages (95% confidence interval)) of colon capsule endoscopy and CT colonography for the detection of lesions, according to size, morphology and histology

	CCE [95% CI]	CTC [95% CI]	***p*** value
Any neoplastic lesion^1^ (regardless of size)	83.2 [78.9–86.9]	42.8 [37.1–48.7]	< 0.001
Any neoplastic lesion^1^ ≥ 6 mm	91.9 [85.6–95.8]	74.8 [66.2–82.2]	0.001
Any neoplastic lesion^1^ ≥10 mm	100 [92.9–100]	88 [76.4–94.6]	0.028
Pedunculated lesions	91.1 [79.4–96.9]	80 [62–90.9]	0.185
Sessile lesions	87.3 [81.8–91.5]	44.2 [36.8–51.7]	< 0.001
Flat lesions	72.6 [63.3–80.3]	20.8 [12.7–31.7]	< 0.001
Cancer	100 [49.9–100]	100 [64.1–100]	–
HGD adenomas	100 [74.4–100]	100 [59.4–100]	–
LGD adenomas	87.1 [81.9–91.1]	46.4 [38.9–54.1]	< 0.001
Serrated lesions	73.6 [61.9–83]	32.9 [22.5–44.9]	< 0.001

*CCE* colon capsule endoscopy, *CTC* CT colonography, *HGD* high-grade dysplasia, *LGD* low-grade dysplasia

^1^Any neoplastic lesion includes cancer, advanced and non-advanced adenomas, and advanced and non-advanced serrated lesions

With respect to histology, there were no differences between both techniques in the detection of cancer or adenomas with high-grade dysplasia, but CCE was able to detect a higher number of adenomas with low-grade dysplasia and serrated lesions than CTC (Table 4).

### Predictors for false-negative results

Independent predictive factors for a false-negative result in CCE examination were lesion size < 6 mm (*p* < 0.001) and inadequate colonic preparation (*p* = 0.05). With respect to CTC, lesion size < 10 mm (*p* < 0.05), caecum or rectum location (*p* < 0.05) and serrated histology (*p* < 0.05) were independent predictors for a false-negative result.

### Adverse events

No serious adverse event was reported in any of the study groups. In the CCE group, a unique mild adverse event was observed in a diabetic patient who presented with vasovagal syndrome after consuming the first prep booster and recovered spontaneously.

## Discussion

This study design allows us to evaluate an enriched population with high prevalence of colorectal neoplasms [5], thus favouring the comparison between CTC and CCE. Results of this randomised trial demonstrate that CCE was more sensitive than CTC in the identification of patients with significant neoplastic lesions (i.e. ≥ 6 mm in size at colonoscopy) (96.1% vs. 79.3%, respectively), which was translated to a higher neoplasm detection rate (98.6% vs. 81.0%, respectively; RR = 1.22; 95% CI, 1.07–1.38). Nonetheless, both techniques identified all patients with CRC and there was no significant difference in the identification of patients with advanced colorectal neoplasms (100% vs. 93.1%, respectively; RR = 1.07; 95% CI, 0.99–1.16) than CTC, the latter without reaching statistical significance. On the contrary, CTC was more specific than CCE (96.3% vs. 88.2%, respectively) in identifying significant neoplastic lesions. Interestingly, these results were observed in both the per-protocol assessment (which was limited to those individuals in whom CCE and CTC were fully evaluable) and the intention-to-screen analysis. In that sense, it is important to mention that CCE could not be evaluated in 18.3% of patients due to incomplete studies. Finally, there were no significant differences in terms of patients’ acceptance and adverse events between both strategies.

There are numerous publications regarding the use of CTC for CRC screening that back up the usefulness of this technique, with sensitivity comparable to conventional colonoscopy. In a seminal article, Pickhardt et al. demonstrated a sensitivity of 94% for lesions ≥ 10 mm and 89% for those ≥ 6 mm [25]. More recent studies confirmed sensitivity figures ranging from 78 to 90% for the detection of these lesions [20, 26, 27]. With respect to CCE, a recent systematic review including over 2000 individuals demonstrate sensitivity values of 87% for lesions ≥ 10 mm and 86% for those ≥ 10 mm, with a specificity of 95% [28]. Recently, a prospective study evaluating CCE in CRC screening showed a sensitivity of 88% for the detection of subjects with significant lesions, with a specificity of 82% [23]. All these figures are very similar to the ones obtained in the present study, thus confirming the reproducibility of our results.

Two studies have previously compared CCE and CTC [29, 30]. Rondonotti et al., including a small number of individuals and performing unblinded colonoscopy, showed a similar diagnostic yield for both techniques [29]. More recently, a second study that focused on compliance was not able to achieve reliable conclusions on polyp detection rate because of the lack of a gold standard in the vast majority of individuals [30].

CCE and CTC represent a two-step approach to CRC diagnosis, in which the first examination selects those individuals who should undergo colonoscopy. In that sense, an adequate balance between sensitivity and specificity of the first examination is critical to minimise the risk of missing lesions and to avoid unnecessarily colonoscopies, respectively. In the present study, both CCE and CTC have shown to be safe and highly effective strategies to detect CRC and advanced adenomas, and therefore, both constitute adequate strategies for CRC diagnosis. Issues referred to medical and technical costs or patient’s preference with respect to these two alternatives should be also ascertained. However, besides logistics or local restrictions favouring the selection of one test over the other, CCE may benefit from a higher sensitivity for detecting any neoplastic lesion with respect to CTC.

Interestingly, our study was the first evaluation comparing the efficacy of CCE and CTC for the identification of serrated lesions (Table 4), which represent an alternative pathway to CRC development. Indeed, these lesions constitute a new challenge to detection at both endoscopy and CTC because of their flat morphology. In the present trial, CCE was superior to CTC in terms of sensitivity for detecting serrated lesions (73.6% vs. 32.9%, respectively; *p* < 0.001), whereas serrated histology was one of the predictors of false-negative results of CTC. These results were reinforced by the fact that CCE detected more sessile and flat lesions than CTC (Table 4), in concordance with the most common presentation of serrated lesions.

No serious adverse event was reported in our trial, in concordance with the results of previous studies [12, 14]. This fact supports that both techniques are safe and, therefore, can be used in a FIT-positive screening setting.

The strength of this study relies on several facts. First, it is the first prospective evaluation of both CCE and CTC performed in a parallel manner and using blinded colonoscopy as the gold standard, which allows determining their specific role in the CRC diagnostic algorithm. Second, the main analysis of results was done by intention-to-screen, thus avoiding the bias of limiting the analysis to those individuals in whom tested examinations were fully evaluable. Third, this study compared CCE and CTC among FIT-positive screenees, an enriched population selected because of its high prevalence of colorectal neoplasms. This circumstance, along with the large sample size, allows an accurate comparison overall, stratified according to colonoscopy findings, and at polyp level.

We are aware, however, of some limitations of the study. First, the use of the above-mentioned enriched population makes it difficult to extrapolate these results to other screening populations. Indeed, the setting of FIT-positive screening population is different in terms of expected yield with respect to one in which CCE or CTC would have been used as the first screening test in naïve individuals. However, the fact that subjects included in the study were selected among participants in a population-based, organised screening programme guarantees an appropriate comparison of the performance of each technique, which is the primary outcome of the study. Second, patients’ acceptance can be overestimated since it has been obtained in a highly motivated population. Nevertheless, this circumstance does not preclude an adequate comparison of subject’s preferences with respect to each option. Third, a higher than expected drop-out rate in both screening strategies may have contributed to the lack of a significant difference when comparing them in terms of advanced neoplasm detection rate. This fact, along with difficulties in the recruitment (47.2% of patients declined to participate due to lack of time to perform both diagnostic tests), resulted in an evaluable study population lower than expected. Fourth, since bowel preparation is a critical issue for both CCE and CTC, it cannot be excluded that results might differ depending on the protocol used. Finally, because of the low number of patients with serrated lesions, it was not possible to analyse performance characteristics of both imaging techniques for each histological subtype (i.e. sessile serrated adenomas/polyps, traditional serrated adenomas and hyperplastic polyps).

## Conclusions

In summary, according to the results obtained in our enriched cohort of FIT-positive individuals, CCE and CTC seem to be equivalent in terms of advanced neoplasm detection rate. However, CCE may benefit from a higher sensitivity for detecting small, flat, sessile and serrated lesions. The impact of this advantage in terms of overall effectiveness deserves further investigations.

## Supplementary information

**Additional file 1: Table S1.** Colon capsule endoscopy preparation. **Table S2.** Baseline characteristics of individuals invited to participate in the study. **Table S3.** Diagnostic performance^1^ of colon capsule endoscopy and CT colonography, according to the per-protocol analysis. **Table S4.** Detection rate^1^ of colon capsule endoscopy and CT colonography, according to the per-protocol analysis.

## Data Availability

Data are available from the corresponding author upon reasonable request.
