# Supplementary material for: Colon capsule endoscopy versus CT colonography in FIT-positive colorectal cancer screening subjects: a prospective randomised trial—the VICOCA study
Source: BMC Med. 2020 Sep 18;18:255. doi: 10.1186/s12916-020-01717-4 (PMC7500543; doi:10.1186/s12916-020-01717-4)
Supplement: Supplementary file 1 — Additional file 1: Table S1. Colon capsule endoscopy preparation. Table S2. Baseline characteristics of individuals invited to participate in the study. Table S3. Diagnostic performance1 of colon capsule endoscopy and CT colonography, according to the per-protocol analysis. Table S4. Detection rate1 of colon capsule endoscopy and CT colonography, according to the per-protocol analysis. [file 12916_2020_1717_MOESM1_ESM.doc]

**Supplementary table 1.** Colon capsule endoscopy preparation.

Day -2:

Pursenid 4 tablets (senosids A+B) before going to bed

Day -1:

Clear liquid diet

7-9 pm: 1 L PEG based solution

Day 0 (exam day):

7-8 am: 1 L PEG based solution

9:30 am: Metoclopramide 10 mg

9:45 AM: CAPSULE ingestion (water + simethicone 80 mg)

After small bowel detection:

- 1^st^ Booster: 500 mL PEG based solution + Gastrografin (50 mL)

- 2^nd^ Booster (3 h after 1st booster): 500 mL PEG based solution + Gastrografin (25 mL)

5 h after 1st booster: Bisacodyl suppository

**Supplementary table 2.** Baseline characteristics of individuals invited to participate in the study

|  | **Accepted** (n=349) | **Declined** (n=313) | p value |
| --- | --- | --- | --- |
| Demographics |  |  |  |
| Age (years-old)^1^ | 60.4 (5.8) | 59.8 (5.7) | pNS |
| Gender |  |  | 0.3 |
| Male | 170 (48.7%) | 165 (52.7%) |  |
| Female | 179 (51.2%) | 148 (47.2%) |  |
| Findings at colonoscopy |  |  |  |
| Invasive cancer |  |  | 0.8 |
| No | 333 (96.2 %) | 287 (95.9 %) |  |
| Yes | 13 (3.7%) | 12 (4.0%) |  |
| Advanced neoplasm^2^ |  |  | 0.3 |
| No | 248 (71.6%) | 224 (74.9 %) |  |
| Yes | 98 (28.3%) | 75 (25.0%) |  |

^1^Continuous variables are expressed as mean (standard deviation).

^2^Advanced neoplasm includes invasive cancer, advanced adenomas, and advanced serrated lesions.

**Supplementary table 3.** Diagnostic performance^1^ of colon capsule endoscopy and CT colonography, according to the per-protocol analysis.

|  | **CCE (%)** | **CI 95%** | **CTC (%)** | **CI 95%** | **Diff.** | **95% CI** |
| --- | --- | --- | --- | --- | --- | --- |
| Any neoplastic lesion^2^ (regardless of size), no. (%) | 95 (80.5)^3^ | | 93 (66.9)^3^ | |  |  |
| Sensitivity | 98.9 | 96.7; 100 | 64.5 | 54.9; 74.4 | 34.4 | [24.0; 44.1] |
| Specificity | 82.6 | 66.6; 96.3 | 95.6 | 88.6; 100 | -13 | [-30.6; 3.8] |
| PPV | 95.9 | 91.6; 99.0 | 96.7 | 91.6; 100 | -0.8 | [-6.9; 5.8] |
| NPV | 95 | 83.3; 100 | 57.1 | 47.0; 68.5 | 37.8 | [20.0; 51.6] |
| Accuracy | 95.7 | 92.3; 99.1 | 74.8 | 68.3; 82.0 | 20.9 | [12.6; 28.9] |
| Any neoplastic lesion^2^ ≥6 mm, no. (%)^4^ | 67 (56.7)^3^ | | 57 (41.0)^3^ | |  |  |
| Sensitivity | 100 | 100; 100 | 80 | 69.0; 90.4 | 19.3 | [8.5; 29.6] |
| Specificity | 86.2 | 75.6; 94.6 | 93.9 | 88.3; 98.6 | -7.6 | [-18.8; 3.2] |
| PPV | 90.5 | 83.3; 96.3 | 90.1 | 81.8; 97.7 | 0.3 | [-10.3; 11.4] |
| NPV | 100 | 100; 100 | 87.5 | 80.0; 94.1 | 12.5 | [4.1; 19.5] |
| Accuracy | 94.1 | 88.9; 97.5 | 88.4 | 82.7; 93.5 | 5.5 | [-1.4; 12.4] |
| Any neoplastic lesion^2^ ≥10 mm, no. (%)^4^ | 35 (29.6)^3^ | | 39 (28.0)^3^ | |  |  |
| Sensitivity | 97.1 | 90.3; 100 | 89.7 | 79.5; 97.6 | 7.4 | [-4.8; 19] |
| Specificity | 93.9 | 88.0; 98.7 | 99 | 96.8; 100 | -5 | [-10.8; 0.7] |
| PPV | 87.1 | 75.0; 97.1 | 97.2 | 91.1; 100 | -10 | [-22.3; 2.9] |
| NPV | 98.7 | 95.8; 100 | 96.1 | 92.0; 99.0 | 2.6 | [-2.5; 7.4] |
| Accuracy | 94.9 | 90.6; 98.3 | 96.4 | 92.8; 99.2 | -1.4 | [-6.7; 3.6] |

CCE, colon capsule endoscopy; CTC, CT colonography; PPV, positive predictive value; NPV, negative predictive value.

^1^All figures are expressed as percentages.

^2^Any neoplastic lesion includes cancer, advanced and non-advanced adenomas, and advanced and non-advanced serrated lesions.

^3^Prevalence of patients with such lesions at colonoscopy.

^4^Lesion size was estimated at colonoscopy.

**Supplementary table 4.** Detection rate^1^ of colon capsule endoscopy and CT colonography, according to the per-protocol analysis.

| **Colorectal lesion^2^** | **CCE** | **CTC** | **RR** | **95% CI** | **p value** |
| --- | --- | --- | --- | --- | --- |
| Cancer | 4 (100%) | 8 (100%) | 1.00 | 1.00-1.00 | - |
| Advanced neoplasm^3^ | 40 (100%) | 40 (93.0%) | 1.07 | 0.99-1.17 | 0.08 |
| Any neoplastic lesion^4^ | 67 (100%) | 46 (80.7%) | 1.24 | 1.09-1.41 | <0.001 |

CCE, colon capsule endoscopy; CTC, CT colonography; RR, relative risk; 95% CI, 95% confidence interval.

^1^The detection rate was calculated as the number of individuals in whom colorectal lesions (i.e. cancer, advanced neoplasm or any neoplasia) were detected in each study arm with respect to the number of patients in whom those lesions were identified at colonoscopy, using a threshold of ≥6 mm in size of lesions detected by either CTC or CCE to indicate the work-up colonoscopy.

^2^Patients were classified according to the most advanced lesion.

^3^Advanced neoplasm includes invasive cancer, advanced adenomas, and advanced serrated lesions.

^4^Any neoplastic lesion includes cancer, advanced and non-advanced adenomas, and advanced and non-advanced serrated lesions.
